# Food consumption and nutrient intake in children on day-care days: results from the nationwide German KiESEL study

**DOI:** 10.3389/fnut.2026.1864533

**Published:** 2026-09-07

**Authors:** Clarissa Spiegler, Nicole Nowak, Maik Döring, Kerstin Clausen, Lisa Walczak, Leonie Burgard, Anna-Kristin Brettschneider, Regina Ensenauer, Thorsten Heuer, Andrea Straßburg, Stefan Storcksdieck genannt Bonsmann

**Affiliations:** 1 Institute of Nutritional Behaviour, Max Rubner-Institut (MRI) - Federal Research Institute of Nutrition and Food, Karlsruhe, Germany; 2 Unit Dietary Exposure and Aggregated Exposure, Department Exposure, German Federal Institute for Risk Assessment, Berlin, Germany; 3 National Reference Centre for Authentic Food, Max Rubner-Institut (MRI)-Federal Research Institute of Nutrition and Food, Kulmbach, Germany; 4 Federal Centre for Daycare and School Meals, Berlin, Germany; 5 Max Rubner-Institut (MRI) - Federal Research Institute of Nutrition and Food, Karlsruhe, Germany

**Keywords:** child care meals, day-care centers, food consumption, meal patterns, nutrient intake, preschoolers, toddlers

## Abstract

**Background:**

Day-care (DC) centers are relevant nutritional environments for young children in Germany. Yet, little is known about food consumption and nutrient intake at DC centers.

**Objectives:**

To characterize meal consumption at DC centers, compare food consumption and nutrient intake on DC days versus non-DC days, and explore the contribution of foods consumed at DC centers to the total daily food consumption (TFC) and nutrient intake.

**Methods:**

The analysis is based on questionnaire and food record data (3 + 1 day) collected within the representative KiESEL study. The present subsample includes 1–5-year-old children with at least one DC day and one non-DC day (*n* = 570). DC days and non-DC days were compared using a mixed linear model.

**Results:**

93.7% of the children ate at least one breakfast and 45.5% ate three meals a day at DC centers regularly. On DC days, more beverages, vegetables, and less meat/sausages and unfavorable foods (e.g., sweets, soft drinks) (as %TFC) were consumed, and intakes of protein (% of energy intake, E%), fiber, calcium (mg/MJ) were higher, while fat intake (E%) was lower than on non-DC days. The diet at DC centers was higher in some favorable foods (e.g., vegetables, fruit, starchy side dishes), but lower in milk/milk products and unfavorable foods, resulting in higher intakes of fiber and lower intakes of mono-/disaccharides and calcium compared to the reference shares (i.e., share of TFC or energy intake at DC centers).

**Conclusion:**

DC days showed more characteristics of a healthy diet than did non-DC days, considerably owing to foods eaten at DC centers. The role of DC centers in Germany as important nutritional environments should be strengthened further by public health measures, e.g., obligatory quality standards for meals at DC centers, targeted support for health-minded public procurement, or increased access to meals at DC centers.

## 1 Introduction

Childhood diet is a key determinant of health throughout life: adequate nutrition ensures the nutrient intake needed for healthy development during childhood (1), and minimizes the risk for disease up until adulthood (2). Furthermore, dietary habits seem to track from childhood into adolescence (3) or even into adulthood (4), where they further influence metabolic health. In Germany, the diet of children aged 1–5 years leaves room for improvement. While virtually all children consume less vegetables than recommended, meat and unfavorable foods (such as sweets and soft drinks) are consumed in excess (5). At the same time, intakes of vitamin D, iodine, iron (in toddlers) and calcium (in preschoolers) are below the reference values, while intakes of saturated fatty acids, free sugars and protein are too high (6, 7). Understanding the various influences on diet quality in childhood is one important step into promoting lifelong health.

One of these influences might be attending day-care (DC) centers. Depending on the length of the daily stay, children may eat one to several meals at DC centers. Those meals impact a child’s food consumption and nutrient intake, but also their eating behavior through eating together with peers (8) and the DC staff (9). For example, certain techniques such as involving children in food preparation in DC centers were found to be associated with increased fruit and vegetable consumption and lower consumption of sweets (9). Studies from other countries showed mixed results of diet quality at DC centers or on DC days. For example, diet on child-care days was lower in added sugars and solid fats in the US (10), with fewer servings of high-fat and high-sugar foods and drinks in child-care than at home (11). In contrast, in Netherlands, the majority of sweet snacks was consumed at DC centers (12). Another example is vegetables, of which children consumed more servings in child-care than at home in the US (11), and Finnish children attending DC consumed vegetables more frequently compared to children cared for at home (13), but in Netherlands, the majority of vegetables was consumed at home (12). On the other hand the majority of fruit was consumed at the DC center in Netherlands (12), but at home in Finland (14). These findings show that DC centers are relevant nutritional environments in childhood and have the potential to positively influence a child’s diet. However, different organizational structures or regulations of communal catering at DC centers limit comparability between countries (15). The limited comparability impedes transferability of the reported results to Germany, even though insights are urgently needed.

In Germany, child day-care is understood as a part of the public welfare system and all children from the age of 1 year have a legal right to publicly funded child-care. This guarantees them either a place in a DC center (*Kita*) or with a registered childminder (*Kindertagespflege*) (16, 17). In 2025, 37.8% of children below the age of 3 years, and 95.0% of children aged 3–6 years attended DC centers (including registered child minders) in Germany (18). Child day-care involves a shared responsibility among the federal government, the federal states, and local municipalities (16). The local municipalities are responsible for the planning, implementation, and the majority of the funding of early childhood education and care services, including the procurement of catering. Each of the 16 federal states in Germany has its own laws or regulations governing early childhood education and care, which includes diet quality standards. While the quality standards for meals in DC centers, developed by the German Nutrition Society (*Deutsche Gesellschaft für Ernährung*, DGE) (19), are voluntary in most federal states, they will become mandatory nationwide by 2030 (20). As nearly all children from age 3 years to school entry age attend DC centers (18), efforts to promote healthy food choices benefit children from all socioeconomic backgrounds. According to results from the German Health Interview and Examination Survey for Children and Adolescents (*Studie zur Gesundheit von Kinder und Jugendlichen in Deutschland*, KiGGS) Wave 2, children from low-income families were found to be more likely to consume soft drinks daily, but less likely to eat fresh fruit (21). Additionally, they were more likely to be overweight or obese (21). Around 15% of children in Germany were at risk for poverty in 2023 (22). Especially for these children, meals provided in DC centers or schools are considered valuable in counteracting poor diet quality (23).

Despite the importance of healthy nutritional environments for young children, little is known about the actual food consumption and nutrient intake in DC centers in Germany. Previous studies from Germany focused on analyzing the offer, e.g., lunch menu plans (24–26) or lunchboxes (27). These studies showed that most lunch menu plans did not fully comply with recommendations. For example, only half of the DC centers limited meat to twice a week, and not all DC centers offered vegetables and salad daily (24, 26). Actual food consumption may differ from the offer and has, to date, not been investigated in Germany. In general, research and preventive measures focus more often on school compared to preschool years (15).

Our analysis aims to close these research gaps on DC centers as important nutritional environments for children in Germany by using data from the representative, cross-sectional Children’s Nutrition Survey to Record Food Consumption (*Kinder-Ernährungsstudie zur Erfassung des Lebensmittelverzehrs*, KiESEL). KiESEL is the first study in Germany to conduct a comprehensive survey of food consumption among toddlers and preschoolers that captures food consumption at DC centers. The objectives of our analysis were to (i) analyze meal consumption at DC centers, (ii) compare food consumption and intakes of selected nutrients on DC days compared to non-DC days, and (iii) explore the contribution of foods consumed at DC centers to the daily food consumption and nutrient intake on DC days.

## 2 Materials and methods

### 2.1 Data assessment

The following analysis is based on data from the representative KiESEL study, carried out by the German Federal Institute for Risk Assessment (*Bundesinstitut für Risikobewertung*, BfR) in 2014–2017. KiESEL is a module of the German Health Examination Survey for Children and Adolescents Wave 2 (*Studie zur Gesundheit von Kinder und Jugendlichen in Deutschland Welle 2*, KiGGS Wave 2) and assessed food consumption of children aged ≥ 6 months to ≤ 5 years living in Germany. The study was originally intended for exposure assessment in the field of food and product safety, which requires representative and detailed food consumption data. Sampling was performed using the gross sample of KiGGS Wave 2, with 167 sample points across Germany. More details on the sampling methods, study design and survey protocol are described by Nowak et al. (28), and Golsong et al. (29). Previous analyses of the KiESEL study include dietary exposure assessments of different contaminants and elements (30, 31), evaluations of dietary supplement intake (32), as well as food consumption and nutrient intake of infants, toddlers and preschoolers (5, 6, 33). The study was approved by the ethics committee of the Berlin Chamber of Physicians (Eth-28/13) and the German Federal Commissioner for Data Protection and Freedom of Information. Written informed consent was given by the primary caregivers of each child enrolled in the study (28, 29). The manuscript was prepared in alignment with the STROBE-nut guidelines (34) (Supplementary Table 1).

In KiESEL, data on food consumption and dietary behavior were collected via weighed food records and a standardized questionnaire, respectively. The total sample includes *n* = 1,104 children. For *n* = 96 children, food record data was missing or not covering at least 3 days (28). Of the remaining, *n* = 118 were infants aged 6–11 months, and *n* = 890 were children aged 1–5 years. The present analysis refers to a subsample of *n* = 570 children aged 1–5 years with at least one DC day and one non-DC day during the protocol period (Supplementary Figure 1). We defined DC days as those days on which the child consumed at least one food or drink item at the DC center. For *n* = 8 children, parents stated in the questionnaire that their child was exclusively cared for at home or at family home care. Since these children attended a DC center on at least one of the protocol days, we assume they changed the type of care during the study, and confirmed this assumption with date information from the weighed food records. These children were excluded from the analysis of the questionnaire due to filter questions (objective i; *n* = 562), but were included in the analysis of the food consumption data (objectives ii and iii; *n* = 570) (Supplementary Figure 1). Age specifications are based on a child’s completed years of life at the beginning of data collection, i.e., “1 year” refers to all children aged 1.0–1.9 years. The present subsample includes *n* = 29 (5.1%) children aged 6 years at the time of data collection, due to the time lag between recruitment and data collection. They were assigned to the group of 5-year-olds in the present analysis.

For objective (i), data were collected using an interviewer-administered standardized questionnaire answered by the parents. The interview was conducted by trained nutritionists during a visit at the participant’s home. Among others, the questionnaire comprised questions on out-of-home consumption, namely which meals the child usually eats at the DC center, which of those meals are usually brought from home and who prepares the lunch eaten at the DC centers. A comprehensive analysis of questionnaire data for the total KiESEL sample is provided by Nowak et al. (35).

For objectives (ii) and (iii), food consumption was assessed via food records on three consecutive days and an independent fourth day, scheduled 2–16 weeks later (3 + 1 design). Per default, a parent-administered weighed food record was used. During the home visit, prior to recording, parents were given a journal with pre-printed log pages, a kitchen scale, and detailed instructions. In the journal, details on the foods and beverages consumed (e.g., type of preparation, brand), the amount consumed, and place and time of consumption were requested (28, 29). Where weighing of foods was not feasible (e.g., out-of-home eating occasions), household measures, information from the packaging, or the KiESEL picture book depicting different portion sizes were utilized to estimate consumed quantities. The KiESEL picture book was developed for the KiESEL study, making use of already existing, validated picture series, e.g., from the EU PANCAKE project (36), and additional silhouettes developed by BfR (29). If foods other than those shown in the picture book were used for estimation, the KiESEL study team recreated and weighed these alternative portion sizes to obtain the respective amounts (35). At DC centers, a simplified estimated food record was used to assess food consumption, requiring a less detailed description of the food items than in the weighed food records. For these DC food records, the KiESEL picture book and household measures were primarily used to estimate the consumed amounts. For foods prepared at home but eaten at DC centers, the parents were instructed to protocol these food items in their own food record to reduce the workload of the caregivers and to get more detailed food descriptions. The caregivers at the DC center were then only requested to specify the corresponding amount eaten in the DC food record. Parent- and DC-administered food records were subsequently combined into one protocol per day. Any ambiguities in protocol entries were clarified by contacting the parents, but not the DC caregivers. In order to improve data accuracy of the DC food records, catering services were asked for their recipes of the recorded meals instead of using standard recipes (28, 29, 35). Human milk quantities (*n* = 13 children) were largely estimated based on the child’s age and feeding frequency. For details on human milk estimates, please refer to Spiegler et al. (5).

During the home visit, the trained staff also measured body height and weight. Subsequently, the BMI was calculated. Following the protocols of the WHO Child Growth Standards for children below 5 years of age (37) and the WHO growth reference for school-aged children and adolescents (38), age- and sex-specific BMI z-scores were calculated. Children were categorized as having overweight, obesity, and underweight in accordance with the cut-off value recommendations by WHO (39), as previously performed by Spiegler et al. (5). Data on the family’s region of residence and SES were collected as part of KiGGS Wave 2 (28). These data were made available for the present analysis. SES categories (low, medium, high) are based on quintiles determined in KiGGS Wave 2 and comprise parental education, occupational status, and income (40). Further information on the assessment of the sociodemographic data is provided elsewhere (28, 29).

### 2.2 Food groups

Each recorded food item was assigned to one food group. The food group classification largely corresponds to that used in previous KiESEL analyses (5), with the exception that bread/cereals and starchy side dishes were analyzed separately here instead of being combined into one superordinate food group (“carbohydrate foods”). For reasons of data skewness, eggs and fish were excluded from the analysis. An overview of the food groups is given in Supplementary Table 2. Composite dishes (e.g., pizza, casseroles) were mostly disaggregated and their ingredients classified separately. For details, please refer to Spiegler et al. (5).

### 2.3 Energy and nutrient intake

To calculate energy and nutrient intake, the protocol items were either linked to the German Food Composition Database (*Bundeslebensmittelschlüssel*, BLS), version 3.02 (41), or to the LEBTAB database (42). LEBTAB is a food composition database containing foods specifically intended for infants and young children, e.g., follow-on formula or meals/purees in jars. In general, all details on the food item provided in the protocol were considered (e.g., preparation method, brand) when linking the food item to the respective database entry. Fortification was considered in the analysis as far as accounted for in the BLS and LEBTAB. Nutrient intake from supplements was not included in this analysis.

### 2.4 Statistics

Descriptive statistics and the analysis of meal consumption in DC centers (objective i) are given in absolute numbers and percentages.

For comparison of food consumption and nutrient intake between the types of days (objective ii), the protocols were separated into those from DC days and from non-DC days. The daily food consumption and nutrient intake per child were then calculated as arithmetic mean of data from all available protocols per type of day, which led to one average DC day and one average non-DC day per child. Non-consumers of a food group were not excluded from the analysis. To avoid bias due to differences in the amount of total food consumption (TFC) or energy intake on DC days and non-DC days, relative units were calculated: for food groups, the contribution to total food consumption (%TFC); for macronutrients, the contribution to energy intake (E%); for fiber and micronutrients, nutrient density (g/MJ or mg/MJ). For analyzing the influence of the type of day (i.e., DC day vs. non-DC day) on food consumption and nutrient intake, a mixed linear model was calculated, adjusting for age, sex, and SES, using the SAS procedure PROC MIXED. Multiple observations per child (i.e., consumption on the two types of days) were considered as random effect in the model. A finding was considered significant if the *p*-value was < 0.05.

As the food consumption on DC days includes both foods eaten at DC centers and at other places, e.g., at home, the contribution of foods consumed at DC centers to the daily food consumption and daily nutrient intake on DC days was investigated (objective iii). For this purpose, the records of DC days were subdivided by place of consumption (i.e., at the DC center vs. at all other places). Food consumption and nutrient intake per place of consumption were calculated per child as mean of data from all available protocols of DC days. Per food group, the mean share of consumption at DC centers was then compared to the mean share of the total food consumption at DC centers, using the Dunnett test in the PROC MIXED procedure to correct for multiple testing. The rationale behind this comparison is that when assuming that the consumption at DC centers and other places were equally composed in terms of food groups, the share consumed at DC center would be similar to that of the total food consumption for each food group. In this way, food groups specific for DC centers are identified. A food group was considered to be consumed at “comparatively” higher or lower amounts when the Dunnett test found a significant difference (*p* < 0.05). For nutrients, the same procedure was used, but the share of nutrients at DC centers was compared against that of energy intake.

Additionally, we conducted a subgroup analysis for objectives (ii) and (iii) to provide further insights into the data and reflect patterns of DC use. For these analyses, we divided the children into those eating lunch at the DC center on a regular basis according to the questionnaire (*n* = 428), and those not having lunch regularly at the DC center (*n* = 142). We assumed having lunch at the DC center to be related to an overall longer stay at the DC center and thus a larger influence on diet quality. The *n* = 8 children without questionnaire data were assigned to the lunch/no-lunch group according to their weighed food records. The statistical methods of objective (ii) (comparison of DC day vs. non-DC day) and objective (iii) (contribution of food eaten at DC centers) were then applied to both groups separately.

For the analysis of questionnaire data, IBM SPSS Statistics Version 26.0.0.1 (IBM Corp., Armonk, NY, United States) was used. For the comparison of DC and non-DC days as well as the contribution of food eaten at DC centers, SAS version 9.4 (SAS Institute, Inc., Cary, NC, United States) was used.

## 3 Results

### 3.1 Sample characteristics

Approximately 70% of the children were aged 3–5 years (Table 1). Only about 5% came from families with low SES. Depending on age group and sex, between 3.3% and 9.5% of the children were living with overweight or obesity. Sociodemographic characteristics of the subgroups (eating lunch at DC center and not eating lunch at DC center) are displayed in Supplementary Table 3.

**TABLE 1:** Sample characteristics of KiESEL children aged 1–2 and 3–5 years^1^, stratified by sex.

Characteristic	1–2 years, *n* = 164		3–5 years, *n* = 406	
	Boys	Girls	Boys	Girls
*n* (%)	90 (54.9)	74 (45.1)	211 (52.0)	195 (48.0)
Socioeconomic status2 (n, %3)				
[1pt] Low	4 (4.4)	0	13 (6.2)	9 (4.6)
Medium	50 (55.6)	40 (54.1)	118 (56.2)	130 (67.0)
High	36 (40.0)	34 (45.9)	79 (37.6)	55 (28.4)
Region of residence4 (n, %3)				
[1pt] North	12 (13.3)	11 (14.9)	23 (10.9)	20 (10.3)
East	37 (41.1)	38 (51.4)	65 (30.8)	64 (32.8)
South	17 (18.9)	17 (23.0)	80 (37.9)	47 (24.1)
West	24 (26.7)	8 (10.8)	43 (20.4)	64 (32.8)
Anthropometric measures (mean ± SD)				
[1pt] Body height (cm)	88.5 (±6.1)	84.6 (±7.4)	107.9 (±7.8)	107.7 (±7.9)
Body weight (kg)	12.9 (±1.8)	12.0 (±2.3)	18.2 (±2.9)	18.2 (±3.3)
Weight classification5 (n, %3)				
[1pt] Underweight	0	1 (1.4)	3 (1.4)	0
Overweight	3 (3.3)	7 (9.5)	16 (7.6)	14 (7.2)
Obese	0	0	0	2 (1.0)

^1^The present sample includes *n* = 570 children with at least one day-care (DC) day and one non-DC day.

^2^For *n* = 2 children, data is missing.

^3^Column %.

^4^Federal states were assigned as follows: North: Schleswig-Holstein, Hamburg, Lower Saxony, Bremen; East: Berlin, Brandenburg, Mecklenburg-Western Pomerania, Saxony, Saxony-Anhalt, Thuringia; South: Baden-Wuerttemberg, Bavaria; West: North Rhine-Westphalia, Hessia, Rhineland-Palatinate, Saarland.

^5^BMI categories were defined as follows: children ≤ 60 months: BMI z-score < −2 SD underweight, >2 to ≤3 SD overweight, >3 SD obesity; children ≥ 61 months: BMI z-score < −2 SD underweight, >1 to ≤2 SD overweight, >2 SD obesity (39).

### 3.2 Meal consumption at DC centers (objective i)

**FIGURE 1:**
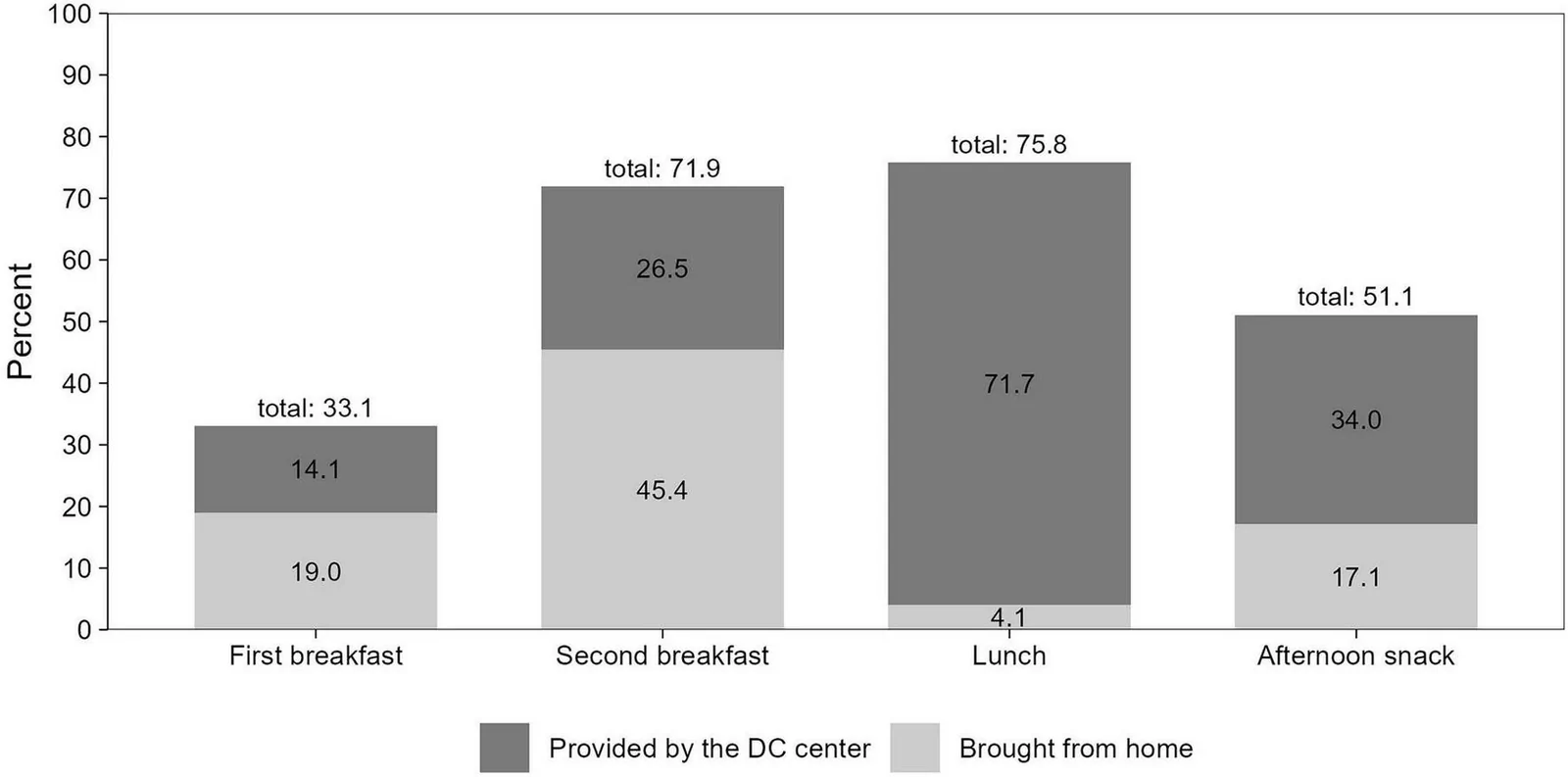
Percentage of children usually consuming the different meals at DC centers, including share provided by DC center and share brought from home (questionnaire data). Small deviations of the total percentage from the sum of the shares are due to rounding. DC, day-care.

The most frequently consumed meal was lunch (Figure 1). Nearly all children (93.6%) usually ate at least one breakfast (first and/or second breakfast) at DC centers (Table 2). Almost 70% ate breakfast and lunch at the DC centers, and little less than half ate breakfast, lunch, and an afternoon snack there. Dinner or late meals were uncommon (0.4%, *n* = 2).

**TABLE 2:** Combinations of meals consumed at DC centers by 1–5-year-old KiESEL children (questionnaire data).

Combinations of meals consumed at the DC center	*n* (%)^1^
At least one breakfast^2^	526 (93.6)
Breakfast^2^ and lunch	391 (69.6)
Breakfast^2^, lunch, and afternoon snack	256 (45.6)

DC, day-care.

^1^% of *n* = 562 children who attended DC centers at the time of the questionnaire interview.

^2^First and/or second breakfast.

Of those consuming the respective meal at the DC center, around half to two thirds typically brought their first and second breakfasts from home, while only one third did so for the afternoon snacks (Figure 1). Lunch, eaten at DC centers by three quarters of the children, was typically provided by either a caterer (70.2% of those children eating lunch) or cooked at the DC center (20.4% of those eating lunch), but only seldom brought from home. 32.2% of the children did not take any meals from home, thus received their food eaten during the stay at the DC center exclusively from the DC center or a caterer.

### 3.3 Food consumption and nutrient intake on DC days and non-DC days (objective ii)

Total daily food consumption and energy intake were higher on DC days compared to non-DC days, when adjusted for age, sex, and SES (+224 g/day and +68 kcal/day, respectively, both *p* < 0.001) (Table 3). Relative to the total food consumption (as %TFC), mean consumption of beverages (+4.1 %TFC) and vegetables (+0.5 %TFC) was higher on DC days, while consumption of meat/sausages (−0.3 %TFC) as well as unfavorable foods (−3.8 %TFC) was lower (all *p* < 0.05). For the other food groups, no significant differences were found. As percentage of energy intake, mean protein intake was higher (+0.5 E%), and mean intakes of fat (−0.9 E%), monounsaturated fatty acids (−0.4 E%), and polyunsaturated fatty acids (−0.3 E%) were lower on DC days than on non-DC days (all *p* < 0.05). Per MJ, mean intakes of fiber (+0.4 g/MJ) and calcium (+5 mg/MJ) were higher on DC days (both *p* < 0.05). For selected other nutrients, no significant differences were found. For absolute values and age stratification, please refer to Supplementary Tables 4, 5, respectively.

**TABLE 3:** Food consumption and nutrient intake of 1–5-year-old KiESEL children on DC days and non-DC days (food record data).

Food group/nutrient	DC days		Non-DC days		*P* ^1^
	Mean	SD	Mean	SD	
Food consumption					
Total food consumption (g/day)	1,583	451	1,359	420	**<0.001**
Beverages (%TFC)	34.6	13.7	30.5	16.6	**<0.001**
Vegetables (%TFC)	5.2	3.9	4.7	4.4	**0.015**
Fruit (%TFC)	15.7	9.2	16.3	11.9	0.292
Starchy side dishes (%TFC)	6.1	4.4	5.9	4.9	0.383
Bread and cereals (%TFC)	5.8	3.5	5.7	3.9	0.716
Milk and milk products (%TFC)	11.7	10.2	12.3	11.4	0.083
Meat and sausages (%TFC)	3.1	2.5	3.4	3.3	**0.016**
Fats and oils (%TFC)	0.7	0.6	0.6	0.7	0.236
Unfavorable foods (%TFC)	13.6	10.3	17.5	14.6	**<0.001**
Energy and nutrient intake					
Energy intake (kcal/day)	1229	294	1161	325	**<0.001**
Energy (MJ/day)	5.1	1.2	4.9	1.4	**<0.001**
Protein (E%)	13.3	2.4	12.8	2.9	**<0.001**
Fat (E%)	32.4	6.9	33.3	7.9	**0.012**
Saturated fatty acids (E%)	15.6	4.0	15.7	4.2	0.746
Monounsaturated fatty acids (E%)	10.7	2.7	11.0	3.3	**0.017**
Polyunsaturated fatty acids (E%)	3.9	1.4	4.1	2.0	**0.004**
Carbohydrates (E%)	53.1	7.2	52.7	8.5	0.329
Mono-/disaccharides (E%)	26.9	8.1	27.5	8.8	0.103
Fiber (g/MJ)	2.6	1.0	2.2	0.8	**<0.001**
Calcium (mg/MJ)	108	39	103	42	**0.005**
Iron (mg/MJ)	1.28	0.37	1.26	0.41	0.140

DC, day-care, E%, contribution to energy intake; %TFC, percentage of total food consumption.

^1^*P*-value for the difference between DC and non-DC days, based on a mixed linear model adjusted for age, sex, and socioeconomic status. Bold: *p*-values < 0.05.

The subgroup analysis largely confirmed these results, but indicates that the differences between DC and non-DC days were related to whether the children eat lunch at the DC center or not: For children regularly eating lunch at the DC center, differences between DC and non-DC days were found for the same food groups and nutrients as for the total sample, and effects pointed into the same direction. For children not eating lunch at DC centers, differences between DC and non-DC days were found only for total food consumption, unfavorable foods, energy intake and fiber, again directed in the same way as for the total sample. Additionally, this group consumed more bread and cereals on DC days (+0.9 %TFC, *p* < 0.05), and had a higher iron intake per MJ (+0.07 mg/MJ, *p* < 0.05) (Supplementary Table 6).

### 3.4 Contribution of consumption at DC centers to total daily consumption or intake (objective iii)

**FIGURE 2:**
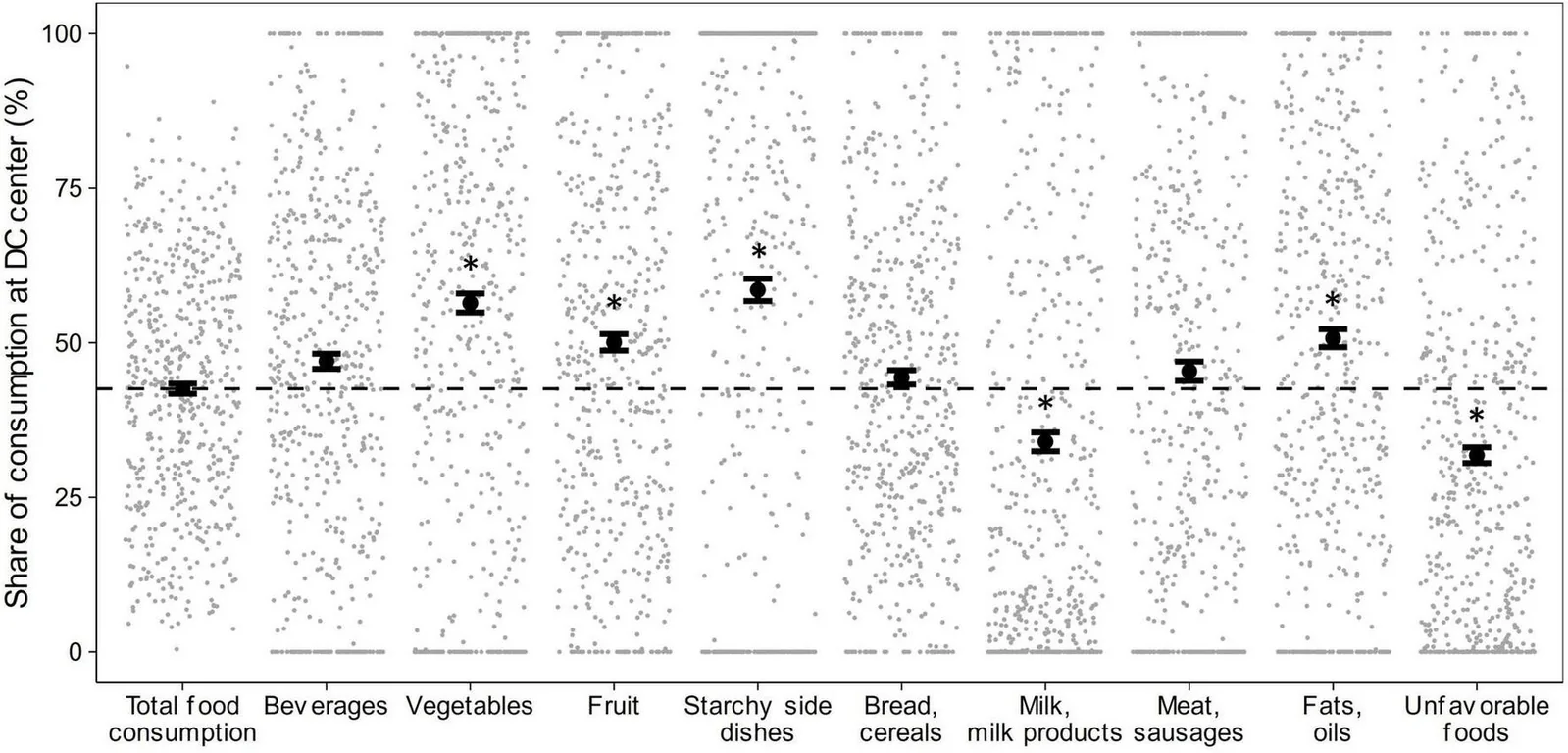
Share of food consumption at DC centers in relation to the respective total daily consumption on DC days in 1–5-year-old KiESEL children (food record data). Dots display individual values. Bold dots and error bars represent means and standard errors of the means. The dotted line represents the mean share of the total food consumption at DC centers in relation to the total daily food consumption on DC days for reference. The asterisks mark food groups with mean shares significantly different to that of the total food consumption, based on the Dunnett test (*p* < 0.05). DC, day-care.

**FIGURE 3:**
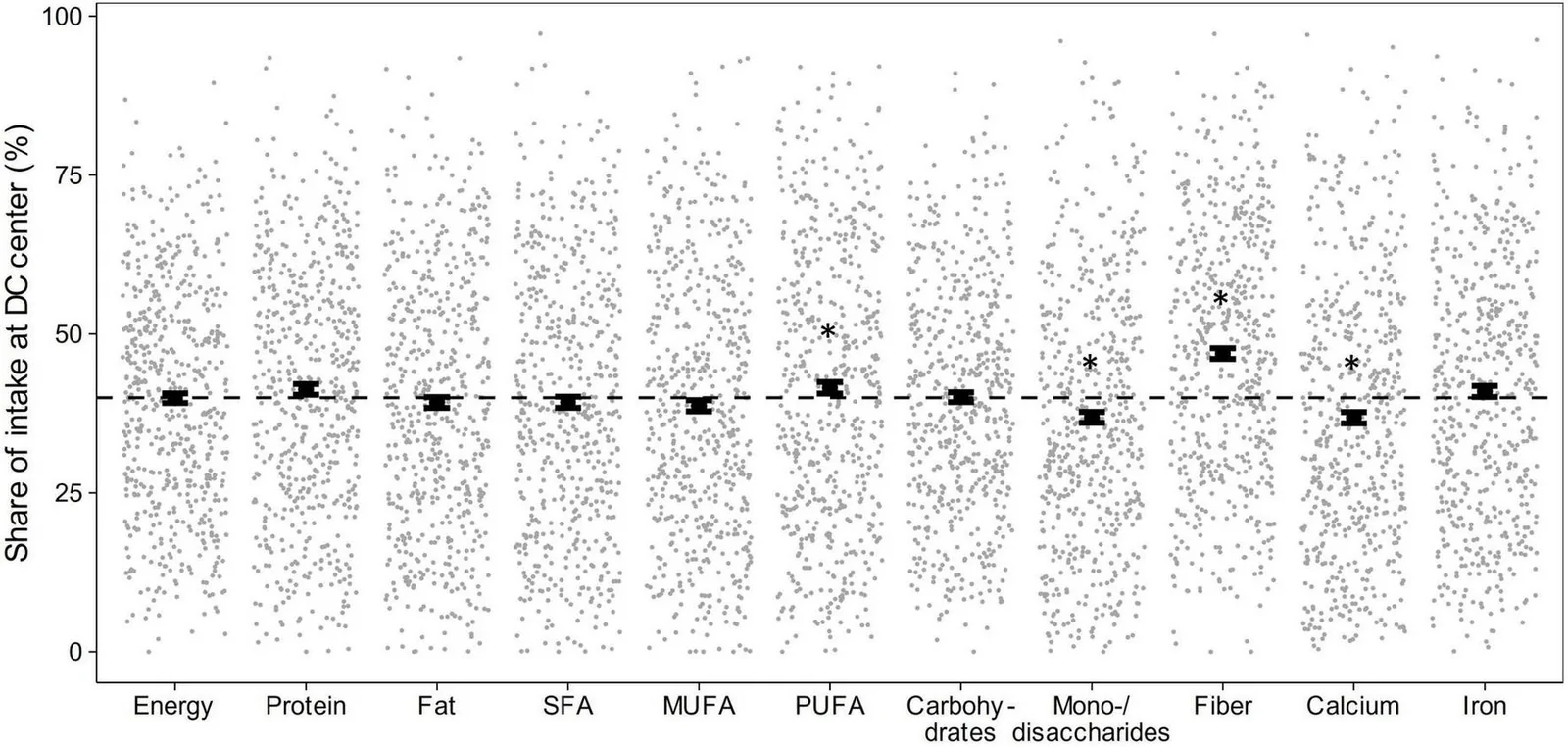
Share of energy and nutrient intake at DC centers in relation to the respective total daily intake on DC days in 1–5-year-old KiESEL children (food record data). Dots display individual values. Bold dots and error bars represent means and standard errors of the means. The dotted line represents the mean share of energy intake at DC centers in relation to the total daily energy intake on DC days for reference. The asterisks mark food groups with mean shares significantly different to that of energy intake, based on the Dunnett test (*p* < 0.05). DC, day-care, MUFA, monounsaturated fatty acids; PUFA, polyunsaturated fatty acids; SFA, saturated fatty acids.

On DC days, food and drinks consumed at DC centers contributed 42.6% of the total daily food consumption and 39.9% of daily energy intake (Figures 2, 3 and Supplementary Table 7). These shares were used as reference in the following comparisons. For the food groups, the share of consumption at DC centers relative to daily consumption ranged between 31.8% and 58.5%. The shares of vegetables (+13.8%-points), fruit (+7.5%-points), starchy side dishes (+15.9%-points), and fats/oils (+8.1%-points) consumed at DC centers were higher, and the share of milk/milk products (−8.6%-points) and unfavorable foods (−10.8%-points) was lower than the reference share of total food consumption at DC centers (all *p* < 0.001). Compared to 39.9% of energy intake, proportional intakes of fiber (+7.0%-points) and polyunsaturated fatty acids (+1.6%-points) were higher at DC centers (*p* < 0.001 and *p* < 0.05, respectively), while the shares of mono-/disaccharides (−3.0%-points) and calcium (−3.1%-points) were lower (both *p* < 0.001).

The subgroup analysis generally confirmed these results for children regularly eating lunch at the DC center. Only the significantly higher fruit consumption was not found in this subgroup, but additionally, the intake of protein was higher than the reference share, i.e., energy intake at the DC center (+2.5%-points, *p* < 0.05). For children not eating lunch at the DC center on a regular basis, the pattern looked differently: The shares of fruit (+14.9%-points) as well as bread and cereals (+15.2%-points) were higher than the share of TFC at the DC center (both *p* < 0.001), but the share of starchy side dishes was lower (−18.6%-points, *p* < 0.001). For the nutrients, a significant difference was only found for fiber intake, which was higher than the reference share (+7.2%-points, *p* < 0.001) (Supplementary Table 8).

## 4 Discussion

The present analysis shows that food consumption at DC centers has a sizable influence on children’s food consumption and nutrient intake. Given that around 40% of the daily food consumption and energy intake took place at DC centers and nearly half of the children regularly ate three meals per day there, excellent diet quality needs to be ensured, which involves implementation as well as regular monitoring. DC days showed more characteristics of a healthy diet (more vegetables and unsweetened beverages, less meat and unfavorable foods, higher fiber and calcium intakes, lower fat intake) compared to non-DC days, although differences were mostly small. The food consumption at the DC center contributed considerably to the daily consumption of some favorable food groups (e.g., vegetables, fruit, starchy side dishes), and at the same time, it was relatively low in milk/milk products and unfavorable foods, resulting in higher intakes of fiber and lower intakes of mono-/disaccharides and calcium. The results point toward DC centers as positively influencing diet quality, more so in children having their lunch regularly at the DC center. This influence affects food groups and nutrients that were previously found to not meet the recommendations in German toddlers and preschoolers, e.g., unfavorable foods, meat as well as free sugar intake (higher than recommended), and vegetables beverages and calcium intake (lower than recommended) (5, 6).

Despite being slightly higher on DC days, vegetable consumption was still below the dietary recommendations on both DC days and non-DC days [5.2 and 4.7 %TFC observed, respectively, vs. 12 %TFC recommended (43)]. The German quality standards for meals in DC centers recommend offering vegetables on a daily basis (19). In a nationwide study, only 6.5% of DC centers followed this recommendation, but most offered vegetables on at least 75% of the days (24). In the present analysis, the share of vegetables consumed at DC centers was comparatively high. Around 70% of this amount was prepared by a caterer or the DC center itself (own unpublished data). This important role of meals at DC centers for vegetable consumption is underlined by results from other surveys: In Finland, a higher vegetable consumption was found in children cared for at DC centers compared to those cared for exclusively at home (13), and in the United States, more servings of vegetables at DC lunch compared to home dinner were observed (11). Furthermore, several studies have shown DC centers to be a suitable setting for interventions to increase vegetable and fruit consumption, e.g., by repeatedly providing vegetables at snack occasions (44, 45), or through gardening interventions (46). Thus, DC centers offer the opportunity to lay the foundation for long-term health by providing healthy meals combined with nutrition education. Considering the overall too low vegetable consumption, this potential needs to be exploited more fully.

The consumption of unfavorable, sugary foods was lower on DC days compared to non-DC days, even in children not eating lunch at the DC center. On DC days specifically, diet at DC centers contributed comparatively less to the daily consumption of unfavorable foods and mono-/disaccharide intake. However, the total consumption of unfavorable foods was still high, also on DC days [28.3 E% observed (data not shown), vs. <10 E% recommended (43)], which urgently needs to be addressed regarding negative health outcomes (47). Around 60% of the amount of unfavorable foods (including desserts, sweet main courses) consumed at the DC center was provided by the DC center or the caterer (own unpublished data), suggesting DC centers to be a good setting to implement public health measures, e.g., mandatory standards for DC meals (20, 47). With most unfavorable foods being consumed outside DC centers, additional measures such as education or taxation of sugar-rich foods are needed to achieve a comprehensive overall reduction in sugar intake (47, 48).

Calcium intake and density were slightly higher on DC days, but the share of calcium intake at DC centers itself was lower than that of energy intake, implying that out-of-DC center calcium intake played a larger role for the total daily calcium intake. Likely, these findings are related to a different meal composition (a) during the course of the day (e.g., milk being mainly consumed with or before the morning meals), and (b) of specific meals on DC and non-DC days (e.g., lunch at the DC center possibly containing more calcium than lunch at home). To verify these hypotheses, further analyses are needed. However, note that the differences, albeit statistically significant, were small. In absolute numbers, the difference between DC and non-DC days was approximately 60 mg/day (Supplementary Table 4), which corresponds to 13% and 7.5% of the dietary reference values for 1–3- and 4–5-year-olds, respectively (49).

Data on the proportion of children eating at DC centers and details on meal provision from other countries are rare (15). Our result that the majority of children (around 75%) eat lunch at the DC center is similar to figures previously reported for Germany (24, 50). The influence of the DC center on diet quality was mainly found for children eating lunch at the DC center, which we assumed to be indicative of an overall longer stay at the DC center. As lunch at the DC center is supposed to provide around 25% of the daily energy and nutrient intake according to the German guidelines (19), this result was expectable. However, some positive influences were also found for children with shorter stays, i.e., having only breakfast at the DC center. For example, the consumption of unfavorable foods was lower on DC days and the intake of fiber was higher. This underlines the potential of optimizing DC meals, even for those attending DC centers only for a few hours.

Notably, a higher energy intake and a higher total food consumption were observed on DC days. This is possibly due to a higher physical activity level at DC centers and on DC days in general (51), and thus higher energy requirements and appetite. However, as no data on physical activity were collected in KiESEL, this hypothesis cannot be verified. As the overall energy intake in KiESEL was in line with the recommendations (6), this difference is not to be rated as critical.

As expected, nearly all DC days were weekdays (>99%, data not shown), and most non-DC days were weekend days in our sample (∼70%, data not shown). In general, dietary behavior differs between weekdays and weekends, e.g., showing higher energy intakes, a higher consumption of added sugars and unfavorable foods, and a lower consumption of fiber and vegetables on weekends (52–56). Such differences might have influenced the presented results. Indeed, consumption on weekdays resembled the consumption on DC days and consumption on weekends resembled that on non-DC days, and a comparison of weekdays to weekend days yielded results similar to the comparison of DC and non-DC days in the present subsample (Table 3 and Supplementary Table 9). Likely, the differences between weekdays and weekend days are linked to different daily routines, e.g., attending DC during the week but not on weekends. Further analyses are needed to better capture the interplay between week/weekend days and DC/non-DC days. Regardless of this, our results demonstrate the important role of the diet at DC centers for a healthy child nutrition, as the proportions of favorable foods and nutrients consumed in DC centers were comparatively large in relation to total food consumption and total energy intake, respectively (Supplementary Table 7).

In general, diet quality at DC centers is influenced by various stakeholders, depending on the structure and size of DC centers: the provider of the DC center is choosing the catering service through public procurement, where quality requirements can be demanded. The chef and kitchen staff or catering service are responsible for the menu plan and a child-oriented meal offer as well as the sensory quality of the foods provided. The educational staff is in charge of accompanying the meals and warranting good general conditions (e.g., enough time, friendly eating environment) to make the meal enjoyable (19). Further, parents may provide food for their child for some meals. Therefore, overall concepts for meals are recommended to ensure that all involved parties follow the same guidelines for a healthy diet (19). Yet, at various stages of the process, obstacles hamper the adoption of high-quality meals at childcare facilities, such as difficulties in formulating the necessary requirements in the public procurement, insufficient resources for monitoring diet quality, difficulties in finding qualified kitchen staff, or budget constraints (57). Joint efforts from all stakeholders as well as support from public health authorities are needed in order to overcome these barriers and ensure excellent diet quality at DC centers.

A key strength of the study are its detailed data on food consumption at DC centers in the context of the whole day, collected for the first time in a nationwide German study sample and thus opening up the opportunity to investigate the contribution of diet at DC centers to the overall diet of toddlers and preschoolers. Feasibility for the DC staff, and hence compliance with the study protocol, was ensured by estimating the amounts via the KiESEL picture book and household measures rather than weighing, and simplifying the protocol after a pretest (28). Almost all of the portion sizes used in the KiESEL picture book were validated, so the estimated amounts are assumed to be as accurate as possible. To compensate for influences of age, sex, and SES on food consumption and nutrient intake, the mixed linear model comparing DC days and non-DC days was adjusted for these parameters. Two aspects limit the generalizability of the results. First, all participants in KiESEL took part in the KiGGS Wave 2 study prior to KiESEL. It is likely that predominantly parents with a strong interest in health and nutrition decided to also participate in KiESEL after having taken part in KiGGS Wave 2. The response rate of KiESEL was 75% (28), suggesting a generally high level of motivation for participating, irrespective of the representative sampling process for the KiGGS Wave 2 sample. Secondly, the proportion of children with low SES was low, and no specific weighting factor to balance out socioeconomic differences from the reference population was available for our subsample.

## 5 Conclusion

The results of our analysis underline the role of DC centers as an important nutritional environment in childhood. DC days in total showed more characteristics of a healthy diet as compared to non-DC days, but still require optimization (e.g., vegetables, calcium). Diet at DC centers was comparatively rich in some favorable and low in unfavorable foods. DC centers thus provide the potential to improve diet quality and support healthy upbringing of all children, independent of their socioeconomic background. Public health measures should aim at exploiting this potential more fully, e.g., through obligatory quality standards for meals at DC centers, targeted support for health-minded public procurement, or increased access to and uptake of meals at DC centers. Additional efforts could seek to promote the transfer of these improved diets to private settings.

## Data Availability

The data analyzed in this study is subject to the following licenses/restrictions: data described in the manuscript (aggregated), code book, and analytic code will be made available upon request pending application and approval. Requests to access these datasets should be directed to Thorsten Heuer, thorsten.heuer@mri.bund.de.
