# Supplementary material for: Food consumption and nutrient intake in children on day-care days: results from the nationwide German KiESEL study
Source: Front Nutr. 2026 Sep 7;13:1864533. doi: 10.3389/fnut.2026.1864533 (PMC13590380; doi:10.3389/fnut.2026.1864533)
Supplement: Supplementary file 1 [file Data_Sheet_1.pdf]

Supplementary Material

Food consumption and nutrient intake in children on day-care days: results from the nationwide German KiESEL study

Table of contents

1 Supplementary Tables 2

Table S1: Reporting checklist for observational studies in nutritional epidemiology – based on the STROBE-nut guidelines (1) 2

Table S2: Food group scheme 6

Table S3: Sociodemographic characteristics, stratified by regular lunch consumption at the DC center 6

Table S4: Food consumption and nutrient intake of 1–5-year-old KiESEL children on DC days and non-DC days (absolute numbers), based on food record data 7

Table S5: Food consumption and nutrient intake on DC days and non-DC days in KiESEL children aged 1–2 years and 3–5 years, based on food record data 8

Table S6: Food consumption and nutrient intake of 1–5-year-old KiESEL children on DC days and non-DC days (food record data), stratified by regular lunch consumption at the DC center 9

Table S7: Food consumption and nutrient intake of KiESEL children aged 1–5 years at DC centers and other places, based on food record data[1] 10

Table S8: Food consumption/nutrient intake at DC center and other places, stratified by regular lunch consumption at the DC center 11

Table S9: Food consumption and nutrient intake on weekdays and weekend days of 1–5-year-old KiESEL children attending DC centers, based on food record data 12

2 Supplementary Figures 13

Figure S1: Flow chart of the study sample 13

3 References 14

1 Supplementary Tables

Table S1: Reporting checklist for observational studies in nutritional epidemiology – based on the STROBE-nut guidelines **(1)**

		Reporting Item	*Section*
**Title and abstract**			
Title	#1a	Indicate the study's design with a commonly used term in the title or the abstract	Title, Abstract
Abstract	#1b	Provide in the abstract an informative and balanced summary of what was done and what was found	Abstract
	#nut-1	State the dietary/nutritional assessment method(s) used in the title or in the abstract.	Abstract
**Introduction**			
Background / rationale	#2	Explain the scientific background and rationale for the investigation being reported	Introduction
Objectives	#3	State specific objectives, including any prespecified hypotheses	Introduction
**Methods**			
Study design	#4	Present key elements of study design early in the paper	2.1
Setting	#5	Describe the setting, locations, and relevant dates, including periods of recruitment, exposure, follow-up, and data collection	2.1
	#nut-5	Describe any characteristics of the study settings that might affect the dietary intake or nutritional status of the participants, if applicable.	**Dietary assessment**: 2.1 **SES and region:** Table 1
Eligibility	#6a	Cohort study: Give the eligibility criteria and the sources and methods of selection of participants. Describe methods of follow-up. Case-control study: Give the eligibility criteria and the sources and methods of case ascertainment and control selection. Give the rationale for the choice of cases and controls. Cross-sectional study: Give the eligibility criteria, and the sources and methods of selection of participants.	n/a, sampling performed within KiGGS Wave 2 (2)
	#6b	Cohort study: For matched studies, give matching criteria and number of exposed and unexposed. Case-control study: For matched studies, give matching criteria and the number of controls per case.	n/a, cross-sectional study
	#nut-6	Report any particular dietary, physiologic, or nutritional characteristics that were considered when selecting the target population.	2.1, Figure S1
Variables	#7	Clearly define all outcomes, exposures, predictors, potential confounders, and effect modifiers. Give diagnostic criteria, if applicable	2.1, 2.2
	#nut-7.1	Clearly define foods, food groups, nutrients, or other food components (e.g., preparation method, taxonomical descriptors, classification, chemical form).	2.2, Table S2
	#nut-7.2	When calculating dietary patterns, describe the methods to obtain them and their nutritional properties.	n/a, no dietary patterns calculated

		Reporting Item	*Section*
Data sources and measurement	#8	For each variable of interest give sources of data and details of methods of assessment (measurement). Describe comparability of assessment methods if there is more than one group. Give information separately for exposed and unexposed groups if applicable.	2.1
	#nut-8.1	Describe the dietary assessment method(s) (e.g., portion size estimation, number of days and items recorded, how it was developed and administered, and how quality was ensured); report if and how supplement intake was assessed.	2.1, 2.3
	#nut-8.2	Describe and justify food-composition data used; explain the procedure to match food composition with consumption data; describe the use of conversion factors, if applicable	2.3
	#nut-8.3	Describe the nutrient requirements, recommendations, or dietary guidelines and the evaluation approach used to compare intake with the dietary reference values, if applicable	n/a, no dietary guidelines or DRVs used for evaluation
	#nut-8.4	When using nutritional biomarkers, additionally use the STROBE-ME; report the type of biomarkers used and usefulness as dietary exposure markers	n/a, no biomarkers used
	#nut-8.5	Describe the assessment of nondietary data (e.g., nutritional status and influencing factors) and timing of the assessment of these variables in relation to dietary assessment	2.1
	#nut-8.6	Report on the validity of the dietary or nutritional assessment methods and any internal or external validation used in the study, if applicable	Described in (3)
Bias	#9	Describe any efforts to address potential sources of bias	Described in (3)
	#nut-9	Report how bias in dietary or nutritional assessment was addressed (e.g., misreporting, changes in habits as a result of being measured, data imputation from other sources).	Refer to (3) (misreporters were not excluded)
Study size	#10	Explain how the study size was arrived at	n/a, sampling performed within KiGGS Wave 2 (2)
Quantitative variables	#11	Explain how quantitative variables were handled in the analyses. If applicable, describe which groupings were chosen, and why	**Day-care/non-day-care days:** 2.1 **Food groups**: 2.1, Table S2
	#nut-11	Explain categorization of dietary/nutritional data (e.g., use of N-tiles and handling of nonconsumers) and the choice of reference category, if applicable.	2.1, 2.4
Statistical methods	#12a	Describe all statistical methods, including those used to control for confounding	2.4
Subgroups and interactions	#12b	Describe any methods used to examine subgroups and interactions	n/a, no subgroup or interaction analyses performed
Missing data	#12c	Explain how missing data were addressed	2.1 (missing food records); no missing

		Reporting Item	*Section*
			food consumption data of included participants
Loss to follow up	#12d	Cohort study: if applicable, explain how loss to follow-up was addressed. Case-control study: if applicable, explain how matching of cases and controls was addressed. Cross-sectional study: if applicable, describe analytical methods taking account of sampling strategy.	n/a, no such methods used
Sensitivity analysis	#12e	Describe any sensitivity analyses	**Subgroup analysis (lunch/no lunch):** 2.4, 3.3, 3.4, Tables S3, S6, S8
	#nut-12.1	Describe any statistical method used to combine dietary or nutritional data, if applicable.	n/a, no data combined
	#nut-12.2	Describe and justify the method for energy adjustments, intake modeling, and use of weighting factors, if applicable	n/a, no such methods used
	#nut-12.3	Report any adjustments for measurement error (i.e., from a validity or calibration study).	n/a, no adjustments performed
**Results**			
Participants	#13a	Report numbers of individuals at each stage of study—eg numbers potentially eligible, examined for eligibility, confirmed eligible, included in the study, completing follow-up, and analysed. Give information separately for exposed and unexposed groups if applicable.	2.1, Figure S1
Non-participation	#13b	Give reasons for non-participation at each stage	n/a, refer to Nowak et al., 2022 (4)
Participant journey	#13c	Consider the use of a flow diagram	Figure S1
	#nut-13	Report the number of individuals excluded on the basis of missing, incomplete, or implausible dietary and nutritional data.	2.1, Figure S1
Descriptive data	#14a	Give characteristics of study participants (eg demographic, clinical, social) and information on exposures and potential confounders. Give information separately for exposed and unexposed groups if applicable.	3.1, Table 1
Missing data	#14b	Indicate number of participants with missing data for each variable of interest	Table 1 (SES), else no missing data
Follow-up time	#14c	Cohort study: Summarise follow-up time (eg, average and total amount)	n/a, cross-sectional study
	#nut-14	Give the distribution of participant characteristics across the exposure variables, if applicable; specify if food consumption for the total population or consumers only was used to obtain results	n/a, all participants attended day-care centers
Outcome data	#15	Cohort study: report numbers of outcome events or summary measures over time. Case-control study: report numbers in each exposure category, or summary measures of exposure. Cross-sectional	**summary measures**: 3.2, 3.3, 3.4, Tables 1–3, Tables S4–S9

		Reporting Item	*Section*
		study: report numbers of outcome events or summary measures.	
Main results	#16a	Give unadjusted estimates and, if applicable, confounder-adjusted estimates and their precision (eg, 95% confidence interval). Make clear which confounders were adjusted for and why they were included	Table 3, Tables S4–S9
Category boundaries	#16b	Report category boundaries when continuous variables were categorized	n/a
Relative and absolute risks	#16c	If relevant, consider translating estimates of relative risk into absolute risk for a meaningful time period	n/a, no RR calculated
	#nut-16	Specify if nutrient intakes are reported with or without the inclusion of dietary supplement intake, if applicable.	2.3
Other analyses	#17	Report other analyses done—eg analyses of subgroups and interactions, and sensitivity analyses	**Food consumption per age group**: Table S5 **Food consumption per lunch/no lunch:** Table S6 and S8
	#nut-17	Report any sensitivity analysis (e.g., exclusion of misreporters or outliers) and data imputation, if applicable	n/a, not performed
**Discussion**			
Key results	#18	Summarise key results with reference to study objectives	4
Limitations	#19	Discuss limitations of the study, taking into account sources of potential bias or imprecision. Discuss both direction and magnitude of any potential bias.	4
	#nut-19	Describe the main limitations of the data sources and assessment methods used and implications for the interpretation of the findings	4
Interpretation	#20	Give a cautious overall interpretation considering objectives, limitations, multiplicity of analyses, results from similar studies, and other relevant evidence.	4, 5
	#nut-20	Report the nutritional relevance of the findings, given the complexity of diet or nutrition as an exposure.	4, 5
Generalisability	#21	Discuss the generalisability (external validity) of the study results	4
**Other Information**			
Funding	#22	Give the source of funding and the role of the funders for the present study and, if applicable, for the original study on which the present article is based	Funding
Ethics	#nut-22.1	Describe the procedure for consent and study approval from ethics committee(s).	Ethics Statement
Data statement	#nut-22.2	Provide data collection tools and data as online material or explain how they can be accessed	Data availability statement

Table S2: Food group scheme

Food group	Examples
Beverages	Water, tea, malt coffee, commercial teas and juices (ready-to-drink products)[1]
Vegetables	Vegetables, legumes, soy and pure soy products (e.g., unflavoured tofu), CCFs with vegetables as main ingredients
Fruit	Fruit, fruit juices, smoothies, CCFs with fruit as main ingredient
Starchy side dishes	Rice, pasta, potatoes and potato products
Bread and cereals	Bread, bread rolls, cereals, cornflakes, muesli, CCFs with cereals as main ingredients
Milk and milk products	Milk, yoghurt, kefir, cream, curd (all non-sweetened), cheese, human milk, milk-based drinks intended for young children, infant and follow-on formula (ready-to-drink products), CCFs with milk as main ingredient
Meat and sausages	Meat, game, poultry, sausages
Fats and oils	Vegetable oils, margarines, butter, lard
Unfavorable foods	Sweets, sweet spreads, ice cream, desserts, cakes, cookies, pancakes, sweetened breakfast cereals, sweetened milk-products (e.g., sweetened fruit yoghurt), salty snack products (e.g., chips, salted nuts), soft drinks, nectars and mixed products, coffee, meat substitutes (excl. pure tofu), plant-based milk alternatives, ready-made sauces (e.g., ketchup), CCFs with unfavorable foods as main ingredients

CCF, commercial complementary foods, i.e., products explicitly labelled for infants and young children (e.g., meals in jars, cereal flakes)
[1] Products specifically labelled for infants and young children

Table S3: Sociodemographic characteristics, stratified by regular lunch consumption at the DC center

	**Lunch** n = 428	**No lunch** n = 142
**Sex** (n, %[3])		
Boys	221 (51.6)	80 (55.3)
Girls	207 (48.4)	62 (43.7)
**Age groups** (n, %[3])		
1–2 years	137 (32.0)	27 (19.0)
3–5 years	291 (68.0)	115 (81.0)
**Socioeconomic status[2]** (n, %[3])		
Low	18 (4.2)	8 (5.6)
Medium	240 (56.3)	98 (69.0)
High	168 (39.4)	36 (25.4)
**Region of residence[4]** (n, %[3])		
North	41 (9.6)	25 (17.6)
East	202 (47.2)	2 (1.4)
South	95 (22.2)	66 (46.5)
West	90 (21.0)	49 (34.5)
**Anthropometric measures (mean ± SD)**		
Body height (cm)	100.8 (± 12.5)	104.4 (± 10.6)
Body weight (kg)	16.4 (± 3.9)	17.2 (± 3.7)
**Weight classification[5] (n, %[3])**		
Underweight	2 (0.4)	2 (1.4)
Overweight	30 (7.0)	10 (7.0)
Obese	2 (0.5)	0

[1] The sample includes n = 570 children with at least one day-care (DC) day and one non-DC day. Subgroups were formed according to the questionnaire answers.
[2] For n = 2 children, data is missing.
[3] Column %
[4] Federal states were assigned as follows: North: Schleswig-Holstein, Hamburg, Lower Saxony, Bremen; East: Berlin, Brandenburg, Mecklenburg-Western Pomerania, Saxony, Saxony-Anhalt, Thuringia; South: Baden-Wuerttemberg, Bavaria; West: North Rhine-Westphalia, Hessia, Rhineland-Palatinate, Saarland
[5] BMI categories were defined as follows: children ≤ 60 months: BMI z-score < -2 SD underweight, > 2 to ≤ 3 SD overweight, > 3 SD obesity; children ≥ 61 months: BMI z-score < -2 SD underweight, > 1 to ≤ 2 SD overweight, > 2 SD obesity (5)

Table S4: Food consumption and nutrient intake of 1–5-year-old KiESEL children on DC days and non-DC days (absolute numbers), based on food record data

	**DC days**		**Non-DC days**	
	Mean	SD	Mean	SD
Beverages (g/day)	570	342	438	322
Vegetables (g/day)	82	66	64	65
Fruit (g/day)	247	164	219	175
Starchy side dishes (g/day)	94	69	77	62
Bread and cereals (g/day)	87	49	74	47
Milk and milk products (g/day)	178	154	161	152
Meat and sausages (g/day)	47	38	46	44
Fats and oils (g/day)	10.6	9.2	8.4	8.9
Unfavorable foods (g/day)	214	174	232	206
Protein (g/day)	41	12	37	13
Fat (g/day)	45	16	44	17
Saturated fatty acids (g/day)	22	9	20	9
Monounsaturated fatty acids (g/day)	15	6	14	6
Polyunsaturated fatty acids (g/day)	5.3	2.5	5.4	3.3
Carbohydrates (g/day)	162	42	152	46
Mono-/disaccharides (g/day)	83	31	79	34
Fiber (g/day)	13.2	7.7	10.4	4.2
Calcium (mg/day)	553	239	490	226
Iron (mg/day)	6.6	2.5	6.1	2.6

Abbreviation: DC, day-care

Table S5: Food consumption and nutrient intake on DC days and non-DC days in KiESEL children aged 1–2 years and 3–5 years, based on food record data

	**1–2 years** n = 164				**3–5 years** n = 406			
	**DC days**		**Non-DC days**		**DC days**		**Non-DC days**	
	Mean	SD	Mean	SD	Mean	SD	Mean	SD
Total food consumption (g/day)	1470	450	1208	416	1629	443	1420	407
Energy intake (kcal/day)	1099	257	974	326	1281	292	1236	292
Energy intake (MJ/day)	4.6	1.1	4.1	1.4	5.4	1.2	5.2	1.2
Beverages (g/day)	503	329	384	323	597	344	460	320
Vegetables (g/day)	71	59	60	73	87	68	65	61
Fruit (g/day)	245	173	199	172	247	161	226	176
Starchy side dishes (g/day)	89	62	68	57	95	71	81	64
Bread and cereals (g/day)	78	48	62	43	91	49	78	48
Milk and milk products (g/day)	217	166	192	166	162	146	148	144
Meat and sausages (g/day)	41	35	35	39	50	39	50	45
Fats and oils (g/day)	10.1	8.9	6.9	8.1	10.8	9.3	9.1	9.2
Unfavorable foods (g/day)	167	160	169	185	233	177	258	208
Beverages (%TFC)	32.8	14.1	29.9	18.1	35.3	13.5	30.8	16.0
Vegetables (%TFC)	4.8	3.6	4.9	5.0	5.4	3.9	4.6	4.1
Fruit (%TFC)	16.6	8.8	16.7	12.3	15.4	9.3	16.1	11.8
Starchy side dishes (%TFC)	6.4	4.4	6.0	5.0	6.0	4.4	5.9	4.8
Bread and cereals (%TFC)	5.7	3.7	5.6	4.2	5.9	3.5	5.8	3.7
Milk and milk products (%TFC)	15.4	11.9	16.6	13.7	10.2	9.1	10.6	9.8
Meat and sausages (%TFC)	2.9	2.6	2.9	3.1	3.1	2.5	3.6	3.3
Fats and oils (%TFC)	0.7	0.6	0.6	0.6	0.7	0.6	0.7	0.7
Unfavorable foods (%TFC)	11.4	9.3	14.2	13.9	14.5	10.6	18.8	14.6
Protein (g/day)	37	10	31	12	42	12	39	12
Fat (g/day)	41	14	36	16	46	17	47	17
Saturated fatty acids (g/day)	20	8	17	8	22	9	22	8
Monounsaturated fatty acids (g/day)	13	5	12	6	15	6	15	7
Polyunsaturated fatty acids (g/day)	4.7	2.1	4.6	3.2	5.6	2.6	5.8	3.3
Carbohydrates (g/day)	143	37	128	45	170	41	162	43
Mono-/disaccharides (g/day)	74	30	68	33	86	31	84	33
Fiber (g/day)	12.0	4.5	9.5	3.8	13.6	8.6	10.8	4.3
Calcium (mg/day)	530	230	451	207	562	243	506	231
Iron (mg/day)	5.9	1.9	5.2	2.3	6.9	2.7	6.4	2.7
Protein (E%)	13.5	2.4	12.8	2.9	13.2	2.4	12.8	2.9
Fat (E%)	33.1	7.0	32.9	7.7	32.2	6.8	33.5	7.9
Saturated fatty acids (E%)	16.0	4.2	15.5	4.5	15.5	3.9	15.8	4.2
Monounsaturated fatty acids (E%)	10.8	2.8	10.8	3.2	10.6	2.7	11.1	3.3
Polyunsaturated fatty acids (E%)	3.8	1.4	4.2	2.3	3.9	1.4	4.1	1.9
Carbohydrates (E%)	52.3	7.1	53.0	8.5	53.4	7.1	52.6	8.6
Mono-/disaccharides (E%)	26.7	8.2	28.1	9.3	26.9	8.1	27.3	8.5
Fiber (g/MJ)	2.6	0.8	2.4	0.9	2.5	1.0	2.1	0.8
Calcium (mg/MJ)	115	42	113	44	105	38	99	41
Iron (mg/MJ)	1.30	0.40	1.30	0.45	1.28	0.36	1.24	0.40

Abbreviations: DC, day-care; E%, contribution to energy intake; %TFC, percentage of total food consumption

Table S6: Food consumption and nutrient intake of 1–5-year-old KiESEL children on DC days and non-DC days (food record data), stratified by regular lunch consumption at the DC center

	**Lunch** n = 428					**No Lunch** n = 142				
	**DC days**		**Non-DC days**			**DC days**		**Non-DC days**		
	Mean	SD	Mean	SD	p[1]	Mean	SD	Mean	SD	p[1]
Total food consumption (g/day)	1610	457	1349	411	**< 0.001**	1502	422	1389	447	**< 0.001**
Beverages (%TFC)	34.6	13.7	29.6	16.7	**< 0.001**	34.5	14.0	33.4	16.0	0.336
Vegetables (%TFC)	5.3	3.8	4.7	4.5	**0.029**	5.0	4.0	4.6	4.1	0.275
Fruit (%TFC)	15.7	9.2	16.8	12.1	0.059	15.8	9.2	14.6	11.2	0.146
Starchy side dishes (%TFC)	6.4	4.4	6.0	4.8	0.163	5.3	4.4	5.7	4.9	0.398
Bread and cereals (%TFC)	5.6	3.3	5.7	4.0	0.264	6.6	4.1	5.7	3.5	**0.015**
Milk and milk products (%TFC)	11.8	10.4	12.4	11.5	0.201	11.3	9.7	12.2	11.0	0.182
Meat and sausages (%TFC)	3.0	2.5	3.4	3.3	**0.013**	3.2	2.5	3.3	3.2	0.617
Fats and oils (%TFC)	0.7	0.6	0.7	0.8	0.252	0.6	0.6	0.6	0.6	0.735
Unfavorable foods (%TFC)	13.3	9.9	17.6	14.7	**< 0.001**	14.6	11.4	16.9	14.0	**0.018**
Energy intake (kcal/day)	1243	304	1171	329	**< 0.001**	1184	257	1130	311	**0.030**
Energy intake (MJ/day)	5.2	1.3	4.9	1.4	**< 0.001**	5.0	1.1	4.7	1.3	**0.030**
Protein (E%)	13.4	2.4	12.8	2.8	**< 0.001**	13.0	2.4	12.9	3.0	0.595
Fat (E%)	32.4	6.9	33.4	8.0	**0.014**	32.4	6.7	32.9	7.5	0.443
Saturated fatty acids (E%)	15.7	4.0	15.7	4.3	0.972	15.3	4.0	15.5	3.9	0.528
Monounsaturated fatty acids (E%)	10.6	2.7	11.1	3.3	**0.006**	10.8	2.8	10.8	3.2	0.960
Polyunsaturated fatty acids (E%)	3.8	1.3	4.1	2.1	**0.003**	4.1	1.6	4.2	1.8	0.568
Carbohydrates (E%)	52.9	7.2	52.6	8.7	0.382	53.4	6.9	53.1	8.1	0.664
Mono-/disaccharides (E%)	26.8	8.2	27.7	8.9	0.074	27.0	7.7	27.0	8.3	0.959
Fiber (g/MJ)	2.6	1.0	2.2	0.8	**< 0.001**	2.4	0.8	2.1	0.7	**< 0.001**
Calcium (mg/MJ)	109	39	103	42	**0.002**	104	38	103	41	0.811
Iron (mg/MJ)	1.30	0.37	1.28	0.41	0.541	1.25	0.36	1.18	0.41	**0.035**

Abbreviations: DC, day-care, E%, contribution to energy intake; %TFC, percentage of total food consumption
[1] p value for the difference between DC and non-DC days, based on a mixed linear model adjusted for age, sex, and socioeconomic status
Bold: p values < 0.05

Table S7: Food consumption and nutrient intake of KiESEL children aged 1–5 years at DC centers and other places, based on food record data[1]

	**Consumption at DC center**		**Consumption at other places**		**Consumption at DC center as share of total consumption (%)**	**p[3]**
	Mean	SD	Mean	SD	Mean[2]	
Total food consumption (g/day)	677	374	906	411	42.6	
Beverages (g/day)	256	200	314	304	47.0	0.053
Vegetables (g/day)	46	52	37	43	56.4	**< 0.001**
Fruit (g/day)	112	95	135	139	50.1	**< 0.001**
Starchy side dishes (g/day)	54	57	39	53	58.5	**< 0.001**
Bread and cereals (g/day)	36	28	51	41	44.4	0.885
Milk and milk products (g/day)	51	75	127	138	34.0	**< 0.001**
Meat and sausages (g/day)	21	25	26	29	45.4	0.602
Fats and oils (g/day)	5.4	6.5	5.3	6.2	50.7	**< 0.001**
Unfavorable foods (g/day)	64	87	149	146	31.8	**< 0.001**
	**Intake at DC center**		**Intake at other places**		**Intake at DC center as share of total intake (%)**	**p[4]**
	Mean	SD	Mean	SD	Mean	
Energy (kcal/day)	492	264	736	279	39.9	
Protein (g/day)	17	10	24	10	41.3	0.114
Fat (g/day)	18	13	27	13	39.2	0.850
Saturated fatty acids (g/day)	8.7	6.6	12.9	6.6	39.3	0.881
Monounsaturated fatty acids (g/day)	5.7	4.3	8.9	4.5	38.7	0.241
Polyunsaturated fatty acids (g/day)	2.2	1.6	3.1	2.1	41.5	**0.034**
Carbohydrates (g/day)	65	34	98	41	40.1	> 0.999
Mono-/disaccharides (g/day)	30	20	53	28	36.9	**< 0.001**
Fiber (g/day)	6.1	3.4	7.1	7.3	46.9	**< 0.001**
Calcium (mg/day)	201	149	352	207	36.8	**< 0.001**
Iron (mg/day)	2.7	1.9	3.9	2.1	41.0	0.357

Abbreviation: DC, day-care

[1] Only consumption on DC days was considered.

[2] The share was calculated on individual level and then averaged. Hence, the share might differ to the ratio of mean consumption at DC centers to the mean consumption at other places.

[3] p value from Dunnett test, comparing the share of each food group against the share of the total food consumption

[4] p value from Dunnett test, comparing the share of each nutrient against the share of the energy intake

Bold: p values < 0.05

Table S8: Food consumption/nutrient intake at DC center and other places, stratified by regular lunch consumption at the DC center

	**Lunch** (n = 428)						**No lunch** (n = 142)					
	**Consumption at DC center**		**Consumption at other places**		**Consumption at DC center (%)[2]**		**Consumption at DC center**		**Consumption at other places**		**Consumption at DC center (%)[2]**	
	Mean	SD	Mean	SD	Mean	p[3]	Mean	SD	Mean	SD	Mean	p[3]
Total food consumption (g/day)	799	340	811	366	49.7		309	181	1192	409	21.0	
Beverages (g/day)	300	201	278	277	54.1	0.140	125	123	423	353	25.7	0.512
Vegetables (g/day)	55	54	31	39	67.0	**< 0.001**	16	28	55	47	21.9	> 0.999
Fruit (g/day)	124	99	127	142	54.7	0.090	74	74	158	126	35.9	**< 0.001**
Starchy side dishes (g/day)	72	55	28	46	75.9	**< 0.001**	1	9	73	59	2.4	**< 0.001**
Bread and cereals (g/day)	38	29	47	37	47.1	0.717	30	26	64	49	36.2	**< 0.001**
Milk and milk products (g/day)	61	81	121	135	39.7	**< 0.001**	20	38	146	146	16.1	0.483
Meat and sausages (g/day)	25	27	22	25	54.1	0.219	8	11	39	36	19.5	0.999
Fats and oils (g/day)	6.4	7.1	4.9	5.8	58.8	**< 0.001**	2.4	3.0	6.3	7.3	25.0	0.652
Unfavorable foods (g/day)	76	93	137	135	37.1	**< 0.001**	30	55	186	171	15.7	0.368
	**Intake at DC center**		**Intake at other places**		**Intake at DC center (%)[4]**		**Intake at DC center**		**Intake at other places**		**Intake at DC center (%)[4]**	
	Mean	SD	Mean	SD	Mean[2]	p[5]	Mean	SD	Mean	SD	Mean[2]	p[5]
Energy (kcal/day)	573	246	671	252	46.0		250	141	935	263	21.5	
Protein (g/day)	20	10	21	9	48.5	**0.002**	7	5	31	10	19.5	0.238
Fat (g/day)	21	13	24	12	45.4	0.970	9	6	34	13	20.7	0.966
Saturated fatty acids (g/day)	10.2	6.7	11.9	6.0	45.1	0.779	4.2	3.2	16.1	7.2	21.6	>0.999
Monounsaturated fatty acids (g/day)	6.8	4.4	8.0	4.1	44.9	0.549	2.8	2.1	11.4	4.8	20.0	0.599
Polyunsaturated fatty acids (g/day)	2.5	1.6	2.8	1.9	48.4	**0.004**	1.1	1.1	4.3	2.3	20.9	0.996
Carbohydrates (g/day)	74	31	89	38	45.8	>0.999	35	22	123	40	22.8	0.780
Mono-/disaccharides (g/day)	34	20	49	27	41.9	**< 0.001**	17	13	64	29	21.7	>0.999
Fiber (g/day)	7.0	3.3	6.6	8.1	52.9	**< 0.001**	3.3	2.0	8.5	3.8	28.7	**< 0.001**
Calcium (mg/day)	235	148	331	207	42.7	**< 0.001**	96	93	416	193	19.1	0.102
Iron (mg/day)	3.1	1.9	3.6	2.1	47.3	0.273	1.3	1.1	4.8	1.9	21.7	>0.999

Abbreviation: DC, day-care

[1] Only consumption on DC days was considered.

[2] As share of the total consumption. The share was calculated on individual level and then averaged. Hence, the share might differ to the ratio of mean consumption at DC centers to the mean consumption at other places.

[3] p value from Dunnett test, comparing the share of each food group against the share of the total food consumption

[4] As share of the total intake. The share was calculated on individual level and then averaged. Hence, the share might differ to the ratio of mean intake at DC centers to the mean intake at other places.

[5] p value from Dunnett test, comparing the share of each nutrient against the share of the energy intake

Bold: p values < 0.05

Table S9: Food consumption and nutrient intake on weekdays and weekend days of 1–5-year-old KiESEL children attending DC centers, based on food record data

	Weekdays		Weekend days		
	Mean	SD	Mean	SD	p[1]
Total food consumption (g/day)	1543	418	1310	472	**<0.001**
Beverages (%TFC)	34.2	13.5	30.0	17.3	**<0.001**
Vegetables (%TFC)	5.1	3.5	4.6	4.5	**0.009**
Fruit (%TFC)	15.9	8.9	16.1	13.2	0.746
Starchy side dishes (%TFC)	6.0	3.8	6.0	5.5	0.898
Bread and cereals (%TFC)	5.7	3.1	5.6	4.1	0.750
Milk and milk products (%TFC)	11.6	9.9	12.5	11.9	**0.019**
Meat and sausages (%TFC)	3.1	2.3	3.6	3.6	**0.001**
Fats and oils (%TFC)	0.7	0.5	0.6	0.7	0.184
Unfavorable foods (%TFC)	14.3	10.6	17.8	15.2	**<0.001**
Energy intake (kcal/day)	1214	281	1128	391	**<0.001**
Protein (E%)	13.1	2.2	12.9	3.2	0.148
Fat (E%)	32.4	6.5	33.6	8.3	**0.002**
Saturated fatty acids (E%)	15.6	3.7	15.8	4.4	0.255
Monounsaturated fatty acids (E%)	10.6	2.6	11.2	3.5	**0.001**
Polyunsaturated fatty acids (E%)	3.9	1.4	4.1	2.1	**0.025**
Carbohydrates (E%)	53.2	6.8	52.3	9.1	**0.018**
Mono-/disaccharides (E%)	27.1	7.6	27.5	9.2	0.287
Fiber (g/MJ)	2.5	0.9	2.2	0.8	**<0.001**
Calcium (mg/MJ)	107	37	103	45	0.052
Iron (mg/MJ)	1.3	0.3	1.3	0.5	0.924

Abbreviations: DC, day-care; E%, contribution to energy intake; %TFC, percentage of total food consumption
[1] p value for the difference between weekdays and weekends, based on the mixed linear model adjusted for age, sex, and socioeconomic status
Bold: p values < 0.05

2 Supplementary Figures

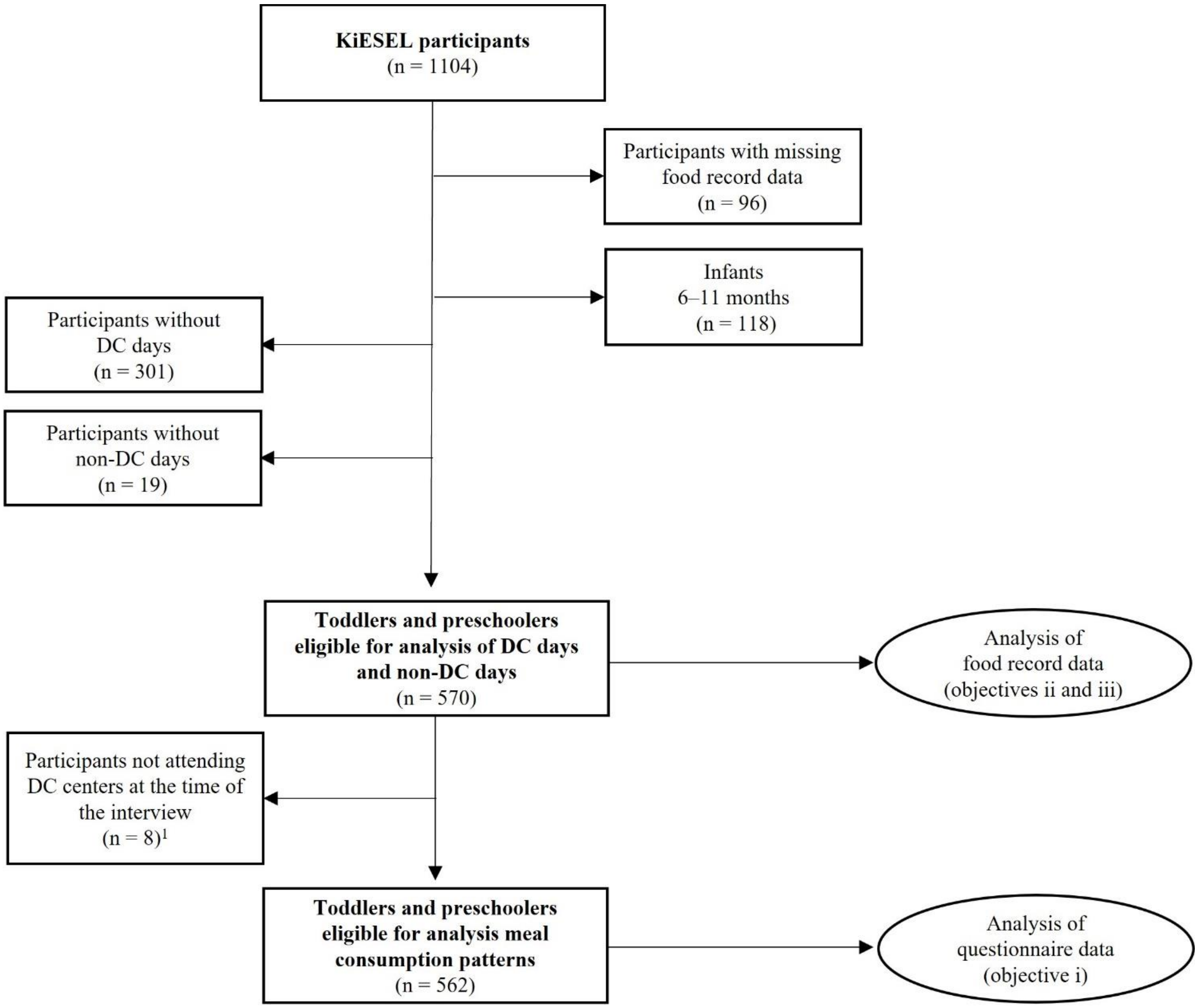

[1] For these children, parents stated in the questionnaire that their child was exclusively cared for at home or at family home care. Since these children attended a DC center on at least one of the protocol days, we assume they changed the type of care during the study, and confirmed this assumption with date information from the weighed food records.

Figure S1: Flow chart of the study sample. DC, day-care

3 References

1. Lachat C, Hawwash D, Ocké MC, Berg C, Forsum E, Hörnell A, et al. Strengthening the Reporting of Observational Studies in Epidemiology - nutritional epidemiology (STROBE-nut): An extension of the STROBE statement. *Nutr Bull* (2016) 41(3):240–51. doi: 10.1111/nbu.12217.
2. Mauz E, Gößwald A, Kamtsiuris P, Hoffmann R, Lange M, von Schenck U, et al. New data for action. Data collection for KiGGS Wave 2 has been completed. *J Health Monit* (2017) 2(Suppl 3):S2–28. doi: 10.17886/RKI-GBE-2017-099.
3. Spiegler C, Jansen S, Burgard L, Wittig F, Brettschneider AK, Schlune A, et al. Unfavorable food consumption in children up to school entry age: results from the nationwide German KiESEL study. *Front Nutr* (2024) 11:1335934. doi: 10.3389/fnut.2024.1335934.
4. Nowak N, Diouf F, Golsong N, Höpfner T, Lindtner O. KiESEL – the children’s nutrition survey to record food consumption for the youngest in Germany. *BMC Nutr* (2022) 8(1):64. doi: 10.1186/s40795-022-00527-6.
5. de Onis M, Lobstein T. Defining obesity risk status in the general childhood population: which cut-offs should we use? *Int J Pediatr Obes* (2010) 5(6):458–60. doi: 10.3109/17477161003615583.
